# Retrospective Study of Hemodynamic Changes Before and After Carotid Stenosis Formation by Vessel Surface Repairing

**DOI:** 10.1038/s41598-018-23842-0

**Published:** 2018-04-03

**Authors:** Xiao Li, Beibei Sun, Huilin Zhao, Xiaoqian Ge, Fuyou Liang, Xuanyu Li, Jianrong Xu, Xiaosheng Liu

**Affiliations:** 1 Department of Radiology, Renji Hospital, School of Medicine, Shanghai Jiaotong University, 160 Pujian Road, Shanghai, China; 2 Shanghai Jiao Tong University and Chiba University International Cooperative Research Center, School of Naval Architecture, Ocean and Civil Engineering, Shanghai Jiao Tong University, Shanghai, China

## Abstract

Prospective observation of hemodynamic changes before and after the formation of atherosclerotic stenosis in the carotid artery is difficult. Thus, a vessel surface repairing method was used for retrospective hemodynamic study before and after atherosclerotic stenosis formation in carotid artery. The three-dimensional geometry of sixteen sinus atherosclerotic stenosis carotid arteries were repaired and restored as normal arteries. Computational fluid dynamics analysis was performed to estimate wall shear stress (WSS), velocity and vortex in atherosclerosis-free areas and sinus in stenosis-repaired carotid artery. The analysis was also performed in the stenotic segment and upstream and downstream of stenosis in stenotic carotid artery. Compared to the atherosclerosis-free areas in stenosis-repaired carotid artery, sinus presented significantly lower WSS (*P* < 0.05), lower velocity (*P* < 0.05) and apparent vortex. Compared to the sinus, the WSS in the upstream of stenosis was lower (*P* < 0.05), while in the downstream area was similar (*P* = 0.87), both upstream and downstream of stenosis demonstrated similar velocity to sinus (*P* = 0.76 and *P* = 0.36, respectively) and apparent vortex. Atherosclerosis-prone areas including normal carotid sinus and upstream and downstream of stenosis in stenotic carotid artery were subjected to lower WSS and velocity as well as apparent vortex, thereby might be associated with the formation and progress of atherosclerosis.

## Introduction

Atherosclerotic disease is the major cause of ischemic stroke or transient ischemic attack^1,2^. The formation and progression of atherosclerosis are caused by complicated pathological process under long-term, complicated hemodynamic actions^3,4^. Thus, understanding the specific hemodynamic factors before the formation of moderate and severe stenosis of the carotid artery is crucial, as stenosis constitutes the major sources of brain embolism^5–7^. Previous studies evaluated the correlation between atherosclerosis and hemodynamic factors in normal and stenotic carotid arteries from different participants^8,9^. However, only limited data are available on the follow-up of the normal carotid artery and atherosclerotic stenosis formation, that prompts a prospective comparative study. Prospective observation of hemodynamic changes during the formation of atherosclerotic stenosis is extremely difficult and often restricted because it is time-consuming and cost-ineffective; also, ethical issues are involved. Therefore, the development of an economic and efficient method to study the hemodynamic changes before and after atherosclerotic stenosis formation is pivotal.

Image-based computational fluid dynamics (CFD) methods can provide blood velocity non-invasively *in vivo*, from which, wall shear stress (WSS) and blood flow patterns can be calculated and analyzed with spatial resolutions exceeding those of the *in vivo* methods^10,11^. Thus, CFD can offer an additional layer of functional information to enrich the anatomical information. Moreover, CFD can virtually remove and repair the lesion segment similar to the normal artery, thereby permitting the analysis of alternative flow scenarios resulting from various states of vessels^12,13^. Therefore, in this study, we used the vessel surface repairing method to virtually remove and repair the carotid sinus stenosis similar to the normal carotid artery. After hemodynamic analysis using CFD in stenotic carotid arteries and stenosis-repaired carotid arteries. Herein, we demonstrated specific hemodynamic features of the stenosis-repaired segment (sinus) and upstream and downstream of stenosis that might provide an in-depth understanding of the atherosclerotic formation and progress in a retrospective study.

## Results

From September 2012 to September 2013, 16 patients with stenotic carotid artery were included. Patients’ characteristics were shown in Table 1.

**Table 1:** Baseline data of stenotic carotid artery patients (n = 16).

Variables	Mean ± SD/n (%)
Age, yr	68.21 ± 13.27
Male gender	10 (62.50%)
BMI, kg/m^3^	24.17 ± 2.39
Previous stroke/TIA	11 (68.75%)
Hypertension	10 (62.50%)
Diabetes	11 (68.75%)
Hyperlipidemia	12 (75.00%)

### Morphology features

A total of sixteen carotid arteries (six left and ten right carotid arteries) with atherosclerotic stenosis were analyzed, of which, twelve had moderate stenosis, and four had severe stenosis. Different components were identified in these plaques. Lipid necrotic core was found in thirteen plaques, calcification was displayed in ten plaques, and hemorrhage was found in six cases. However, no ulcer or thrombus was found in these plaques. Five carotid arteries underwent carotid artery stenting, and the other eleven patients treated with medication only.

### Hemodynamic features

#### Hemodynamic features of normal carotid artery

**Figure 1:**
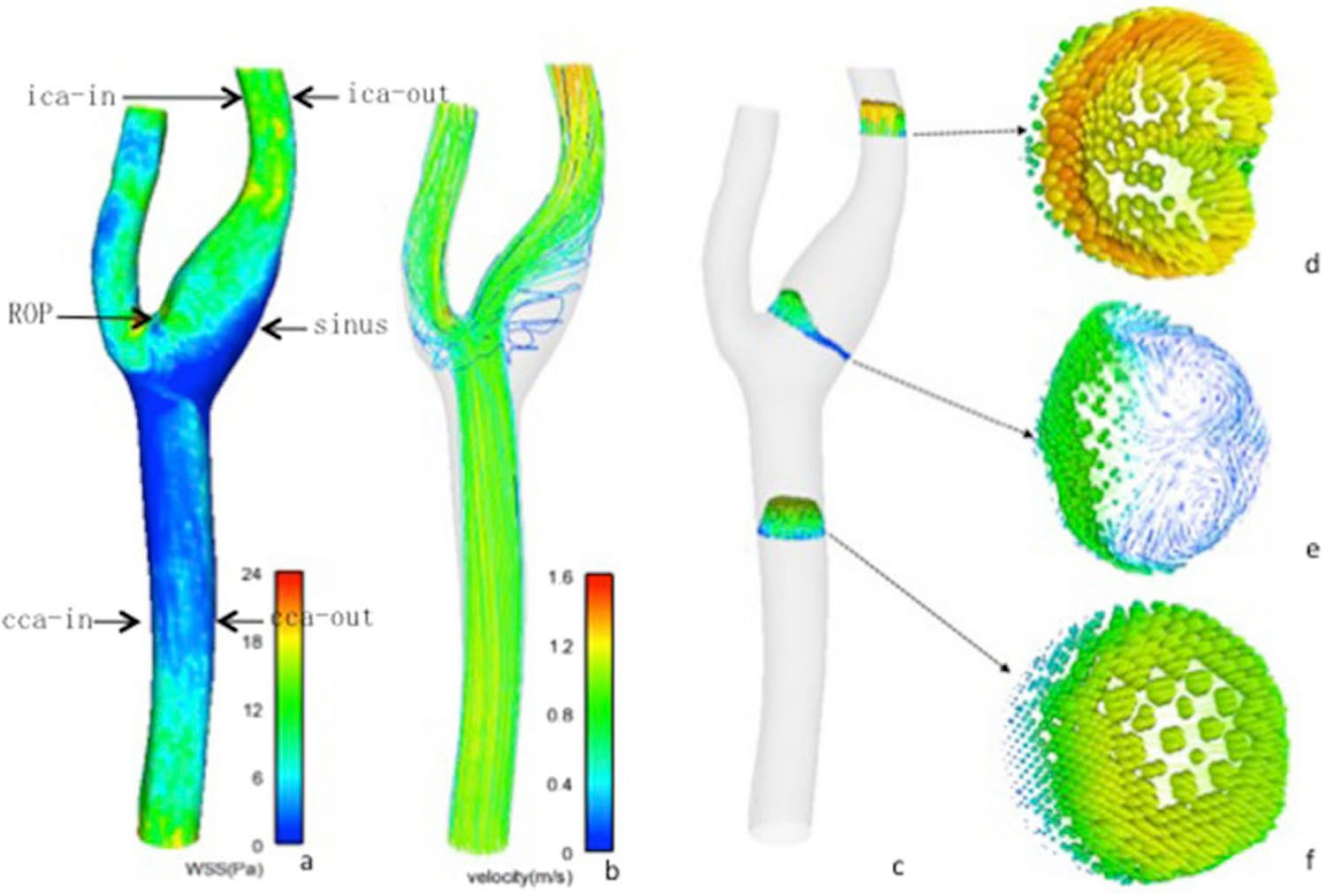
Distribution of WSS **(a**) and velocity (**b–f**) in the normal carotid artery. The sinus featured with low WSS value was indicated in dark blue. Velocity streamlines and axial cut planes with in-plane velocity vectors in normal carotid artery represented the laminar flow started from CCA (**f**) and impinged to the bifurcation, changed its direction, slowed down and formed a vortex in the sinus (**e**), and returned to the laminar flow at ICA (**d**).

In normal carotid artery, the carotid sinus manifested significantly lower WSS than on ROP and outer and inner lateral wall at CCA and ICA level (Fig. 1a). The sinus also featured significantly lower velocity than on ROP and at the CCA and ICA level (Fig. 1b–f). In the sinus, the direction of the flow was altered for vortex formation (Fig. 1e).

#### Hemodynamic features of stenosis-repaired carotid artery

**Figure 2:**
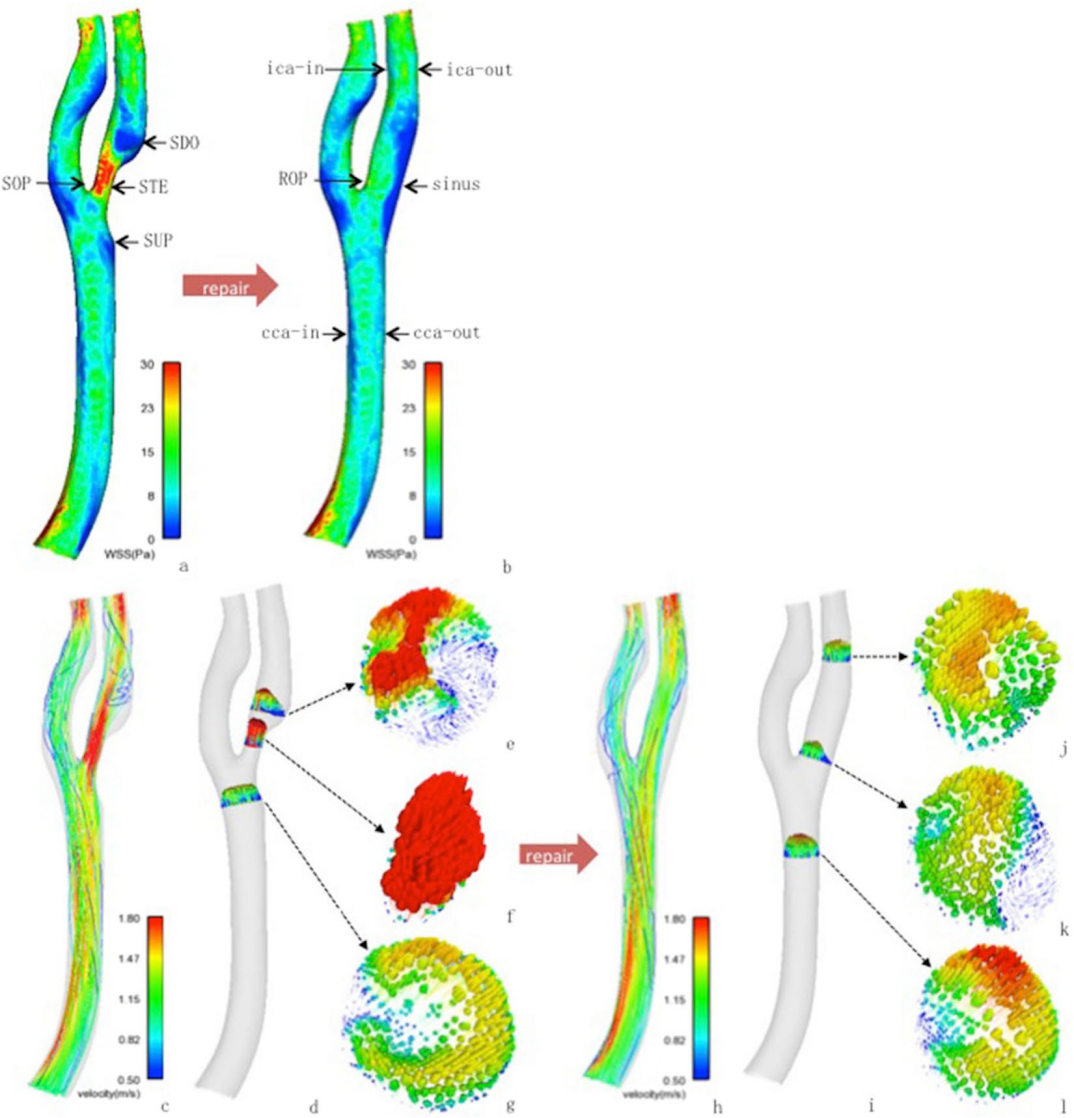
Distribution of WSS and velocity in a stenotic carotid artery and stenosis-repaired carotid artery. The zones characterized with low WSS value were indicated in the dark blue, and thus, presumed to be susceptible to atherosclerosis formation and development. Velocity streamlines and axial cut planes with in-plane velocity vectors in stenosis-repaired carotid artery represented the laminar flow started from CCA (l) and impinged to the bifurcation, changed its direction, slowed down and formed a vortex in the sinus (**k**), and returned to laminar flow at ICA (**j**). Velocity vectors in **k** of the sinus are depicted with low velocity (blue and short vectors) and vortex in this region. Velocity streamlines and axial cut planes with in-plane velocity vectors in stenotic carotid artery represented low velocity (blue and short vectors) and vortex in SUP (**g**), sped up in STE (**f**), and formed low velocity (blue and short vectors) and vortex in SDO (**e**).

In stenosis-repaired carotid artery, carotid sinus had significantly lower WSS than on ROP and outer and inner lateral wall at the CCA and ICA level (Table 2, Fig. 2b). The sinus had significantly lower velocity than on ROP and at the CCA and ICA level (Table 2, Fig. 2h–l). The flow in sinus slowed down and formed the vortex (Fig. 2k).

**Table 2:** WSS and Velocity in stenosis-repaired artery: sinus compared with atherosclerosis-free areas (ROP, cca-out, cca-in, ica-out, ica-in). median (P25-P75).

	WSS (Pa)	*P*	Velocity (m/s)	*P*
	sinus vs. atherosclerosis-free area		sinus vs. atherosclerosis-free area	
ROP	4.66(3.13–6.25) vs. 14.30(12.90–20.13)	<0.001	0.43(0.18–0.65) vs. 1.20(1.08–1.43)	<0.001
cca-out	4.66(3.13–6.25) vs. 6.51(5.55–7.02)	<0.001	0.43(0.18–0.65) vs. 0.94(0.78–1.03)	<0.001
cca-in	4.66(3.13–6.25) vs. 7.05(5.05–8.53)	0.014	0.43(0.18–0.65) vs. 0.79(0.61–0.90)	<0.001
ica-out	4.66(3.13–6.25) vs. 12.20(7.32–18.05)	<0.001	0.43(0.18–0.65) vs. 0.91(0.38–2.04)	<0.001
ica-in	4.66(3.13–6.25) vs. 8.71(6.24–23.13)	<0.001	0.43(0.18–0.65) vs. 1.29(0.90–1.88)	<0.001

#### Hemodynamic features of stenotic carotid artery

For stenotic carotid artery, the STE located in the sinus had significantly increased WSS as compared to the counterpart (sinus) in the stenosis-repaired carotid artery (Fig. 2a). The velocity was significantly increased at STE as compared to the corresponding sinus in the stenosis-repaired carotid artery (Fig. 2f) (Table 3).

**Table 3:** WSS and velocity in STE in stenotic carotid artery compared with sinus in stenosis-repaired carotid artery. median (P25-P75).

	STE vs. sinus	*P*
WSS (Pa)	23.80(14.54–27.13) vs.4.66 (3.13–6.25)	<0.001
Velocity (m/s)	1.33(1.06–1.76) vs.0.43 (0.18–0.65)	<0.001

#### Hemodynamic features at the upstream and downstream of stenosis

The upstream and downstream of stenosis in stenotic carotid artery in comparison to the stenosis-repaired carotid artery revealed low WSS and velocity (Table 4). The WSS at SUP was significantly lower than sinus in the stenosis-repaired artery. The WSS at SDO did not differ significantly from the sinus in the stenosis-repaired artery. The velocity at SUP and SDO was not significantly different as compared to a sinus in the stenosis-repaired artery. Both SUP and SDO developed the vortex. In conclusion, both SUP and SDO were characterized by low WSS and low velocity with a vortex that was similar to the sinus (Fig. 2a and c–g).

**Table 4:** WSS and velocity in SUP and SDO of stenotic carotid artery compared with sinus in stenosis-repaired carotid artery. median (P25-P75).

	WSS (Pa)	*P*	Velocity (m/s)	*P*
SUP vs. sinus	3.44(1.98–4.64) vs.4.66 (3.13–6.25)	<0.001	0.43(0.24–0.68) vs. 0.43(0.18–0.65)	0.762
SDO vs. sinus	5.16(1.86–11.83) vs. 4.66(3.13–6.25)	0.873	0.21(0.11–0.92) vs. 0.43(0.18–0.65)	0.357

#### Formation of further stenosis

**Figure 3:**
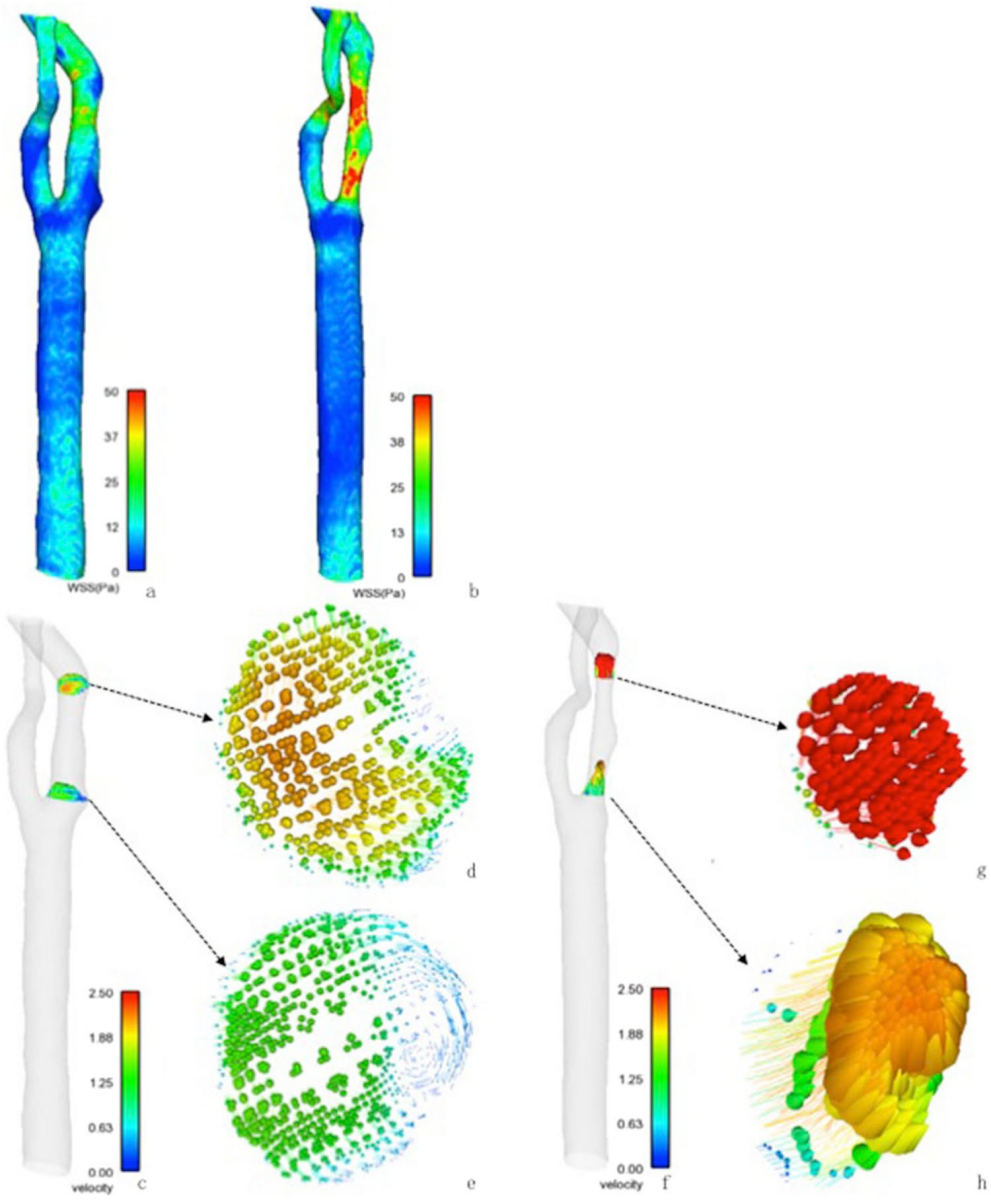
Distribution of WSS and velocity at a follow-up case for four years. An 80-year-old male with hypertension for 12 years and diabetes for 9 years had frequent attacks of dizziness and blurred vision in December 2012; CE-MRA revealed atherosclerotic stenosis in the left proximal ICA. In February 2016, he had a transient ischemic attack and exacerbated stenosis was demonstrated in the left carotid artery by CE-MRA. WSS on the wall of the stenotic carotid artery of 2012 (**a**) and 2016 (**b**). The upstream and downstream of stenosis in a stenotic artery of 2012 characterized by low WSS value was indicated in blue, and thus, presumed to be susceptible to the further development of atherosclerosis. (**b**) Showed the progression of atherosclerotic plaque in the upstream and downstream area of stenosis in the stenotic artery of 2012. Velocity axial cut planes with in-plane velocity vectors in the stenotic artery of 2012 (**c**–**e**) and 2016 (**f**–**h**) represented low velocity (blue and short vectors) and vortex in upstream (**e**) and downstream (**d**) of stenosis in 2012 and sped up in counterparts (**h** and **g**) in 2016.

We followed up one case (of sixteen) for 4 years to detect the next location for further stenosis. In 2012, WSS at the SDO and SUP was lower than that on the walls of the surrounding areas (Fig. 3a). At the SDO and SUP, the velocity was significantly lower with a vortex in the outer lateral layers than the surrounding areas (Fig. 3c–e). The SDO and SUP areas of the carotid artery (2012) showed the progression of plaques in 2016 accompanied by significantly increased WSS and velocity (Fig. 3b and f–h). The significant stenosis progression in SDO and SUP in the carotid artery (2012) supported the result that the stenosis might progress at the areas with low WSS, low velocity, and vortex.

## Discussion

The present study used vessel surface repairing method for a retrospective hemodynamic analysis in carotid artery before and after atherosclerotic stenosis formation. We found that (1) the carotid sinus in stenosis-repaired carotid artery was subjected to low WSS and low velocity, as well as, apparent vortex and (2) hemodynamic analysis at stenotic carotid artery showed that the upstream and downstream of stenosis were subject to extremely low WSS, low velocity, and apparent vortex.

A previous study demonstrated that the sinus in normal carotid artery featured low WSS, low velocity, and apparent vortex. The stenosis-repaired carotid arteries exhibited characteristics similar to the normal carotid artery. To the best of our knowledge, this is the first study that investigated the hemodynamic parameters in atherosclerotic stenosis and stenosis-repaired carotid artery by CFD based on three-dimensional CE-MRA data for longitudinal research. The data modeling of the target vessel is the initial step for hemodynamic analysis. Recently, CFD-based analysis of atherosclerosis has become a crucial method for modeling the vessel and investigating the mechanism underlying atherosclerotic formation and progression. CFD allows for an *in vivo* assessment of individual hemodynamics with high reproducibility^14^. In addition to the visualization of complex three-dimensional blood flow patterns at any ROI in the vessel, CFD can virtually remove and repair the lesion segment back to the nearly normal artery^12,13^. A precise recovery of the atherosclerotic artery is vital. We used the mean and variance curvature calculation method to determine the filling data in the missing area based on the complex of the boundary contour of the vessel model. Then, the calculated data in the atherosclerotic area was used to construct a continuous surface that was similar to the original surface^13^. Gao *et al*.^12^ used the vessel surface repairing method to remove an aneurysm virtually and reconstructed the surface to simulate the normal basilar bifurcation and compared to the basilar arteries with and without an aneurysm by CFD. Similarly, we virtually removed the stenosis and restored to the nearly normal carotid artery. Thus, the sinus in the stenosis-repaired carotid arteries truly developed the atherosclerotic plaque for analysis without the influence of geometry and clinical factors. Using this novel approach, we compared the hemodynamic parameters between stenosis-repaired and stenotic carotid artery similar to the longitudinal research and observed the effect of the hemodynamic features on atherogenesis and atherosclerosis progression.

CFD revealed that low WSS, low velocity, and vortex might be associated with atherogenesis at the stenosis-repaired carotid artery. Areas of the artery with uniform geometry, such as the CCA were exposed to an uninterrupted, unidirectional flow, which exerted a physiological WSS. With the bifurcation and expansion of vessel in the sinus, the flow skews and shifts towards the inner wall of the ICA, and therefore sinus is exposed to low and disturbed flow^15–17^. The disturbed flow with vortex resulted in an energy loss in the sinus, leading to a reduction in the flow velocity and WSS^18^. Several mechanisms have been proposed to elucidate the association between low WSS and atherogenesis, including the modulation of endothelial function and structure, regulation of gene expression, modification of bulk transport of lipids, and promotion of monocyte adhesion to the endothelial wall^19–21^. This finding was in accordance with those of previous studies. Consequently, a reverse study via vessel surface repairing method of the diseased artery may be applied as a prospective study.

Furthermore, the progressive change in the hemodynamic profile with atherosclerotic plaque formation was observed. Downstream to stenosis, a fundamental shift in hemodynamics, with low WSS, low velocity, and the vortex was noted. Huang *et al*.^22^ found that plaque was initiated in the sinus and progressed downstream as analyzed in thirty patients with different stages of stenosis. Michael *et al*.^8^ compared seventeen stenotic carotid arteries with sixty-four normal carotid arteries and found that the presence of moderate-proximal ICA stenosis distinctly altered the low WSS to move distally to the ICA plaque. Birchall *et al*.^23^ observed the low WSS, velocity, secondary turbulence, and recirculating flows downstream to the stenosis with respect to progression. The current study was in agreement with above studies that low WSS, low velocity, and vortex distribution at the downstream contributed to the plaque development on the distal side. In addition, we found that the upstream side was also characterized by low WSS, low velocity, and apparent vortex. The follow-up case showed that the further stenosis was not only located in SDO but also SUP. Sousa *et al*.^14^ revealed a low WSS value in the sinus region, as well as upstream and downstream of stenosis. However, only one patient with bifurcation stenosis was included in the study. Herein, we extended and supported their conclusion with sixteen stenotic carotid arteries.

The present study has several limitations. The main limitation of this study was that the vessel surface repairing method only made the stenosis-repaired carotid arteries normal-like rather than true normal ones. In addition, although vessel wall repairing method had been applied in aneurysms^12,13^, there was rarely applied in stenotic carotid artery. The feasibility of this method needs to be confirmed. The second limitation was that although the present atherosclerotic and hemodynamic analyses potentiate some factors for the diffused progression of carotid artery disease, the natural progression history of carotid atherosclerosis associated with hemodynamic factors may be clarified when carotid arteries are examined by the subsequent follow-up studies in the same patients. The last limitation was that in elucidating the hemodynamic parameters role in the progress of plaque, only the plaques exist or not was considered. Further studies with various stenosis degrees, volume, and additional factors are imperative.

## Conclusions

The sinus in the stenosis-repaired artery and upstream and downstream of stenosis in stenotic artery harbor a hemodynamic environment characterized by low WSS, low velocity, and apparent vortex, might be associated with the formation and progression of atherosclerosis. Herein, the hemodynamic study of virtually repaired carotid arteries by CFD seems a promising method. These results can support further experimental studies investigating the multifactorial driving forces accompanied by the hemodynamic factor underlying the initiation and progression of carotid atherosclerosis.

## Methods

### Ethics approval of the study protocol

This study was conducted in accordance with the Declaration of Helsinki and approved by the Ethics Committee of the Renji Hospital of Shanghai Jiao Tong University with signed informed consent obtained from all subjects.

### Patient and angiographic data

The clinical and imaging data of carotid arteries with sinus moderate and severe atherosclerotic stenosis (stenosis degree ≥30%) from sixteen subjects were collected between September 2012 and September 2013. Stenosis degree was defined according to the North American Symptomatic Carotid Endarterectomy Trial criteria, with the following equation: (1 − LD_min_/RD) × 100, where LD_min_ is minimum lumen diameter and RD is reference diameter and the minimum lumen diameter was defined as the narrowest diameter of the stenotic lesion, measured in the direction perpendicular to the artery, and the reference diameter was defined as the normal diameter distal to the carotid artery lesion^24,25^. In addition, twelve patients with sixteen normal carotid arteries as normal controls were enrolled in the study. All these subjects underwent contrast-enhanced magnetic resonance angiography (CE-MRA) on a 3.0 T MR scanner (Achieva, Philips Healthcare, Best, the Netherlands) using an eight-channel phased-array carotid artery coil (Shanghai Chenguang). The following parameters were set: slice thickness, 2 mm; repetition time (ms)/echo time (ms), 5.1/1.69; field of view, 25.0 × 15.9 cm; acquisition matrix, 250 × 159; acquisition matrix, 1 × 1 mm; acquisition time, 39s. The three-dimensional CE-MRA data of the stenotic and normal carotid arteries in these subjects were output as digital imaging and communications in medicine (DICOM).

### CFD analysis

The three-dimensional CE-MRA data were analyzed by Amira 5.2.2 software (Visage Imaging, San Diego, CA, USA). The stenotic segment of the carotid arteries was repaired and restored to nearly normal carotid sinus using the MeshLab software (version 1.2.2, Visual Computing Lab, ISTI, CNR, Toscana, Italy). All the stenotic arteries, stenosis-repaired arteries, and normal arteries were generated by hybrid, predominantly high-resolution hexahedral mesh using Harpoon (version 4.3 SHARC Ltd, Manchester, UK). A typical mesh contained approximately 1000,000 cells with an entrance length measured at least ten vessel diameters proximal to the carotid bifurcation in order to ensure the development of a Womersley flow profile^26^. Blood flow in the three-dimensional reconstructed models was simulated based on the unsteady incompressible Navier–Stokes equations using the finite-volume method with Ansys Fluent software (version 6.3.26 Ansys, Lebanon, NH, USA). The inflow boundary condition of each model was defined at the inlet of the common carotid artery (CCA). We imposed time-varying velocity profile at the CCA inlet based on patient-specific flow waveform measured by ultrasound. All hemodynamic parameters were measured at the peak of the systolic phase. The vascular wall was presumed to be rigid, and velocity conditions obeyed the nonslip and nonpenetration constraints posed by the wall.

**Figure 4:**
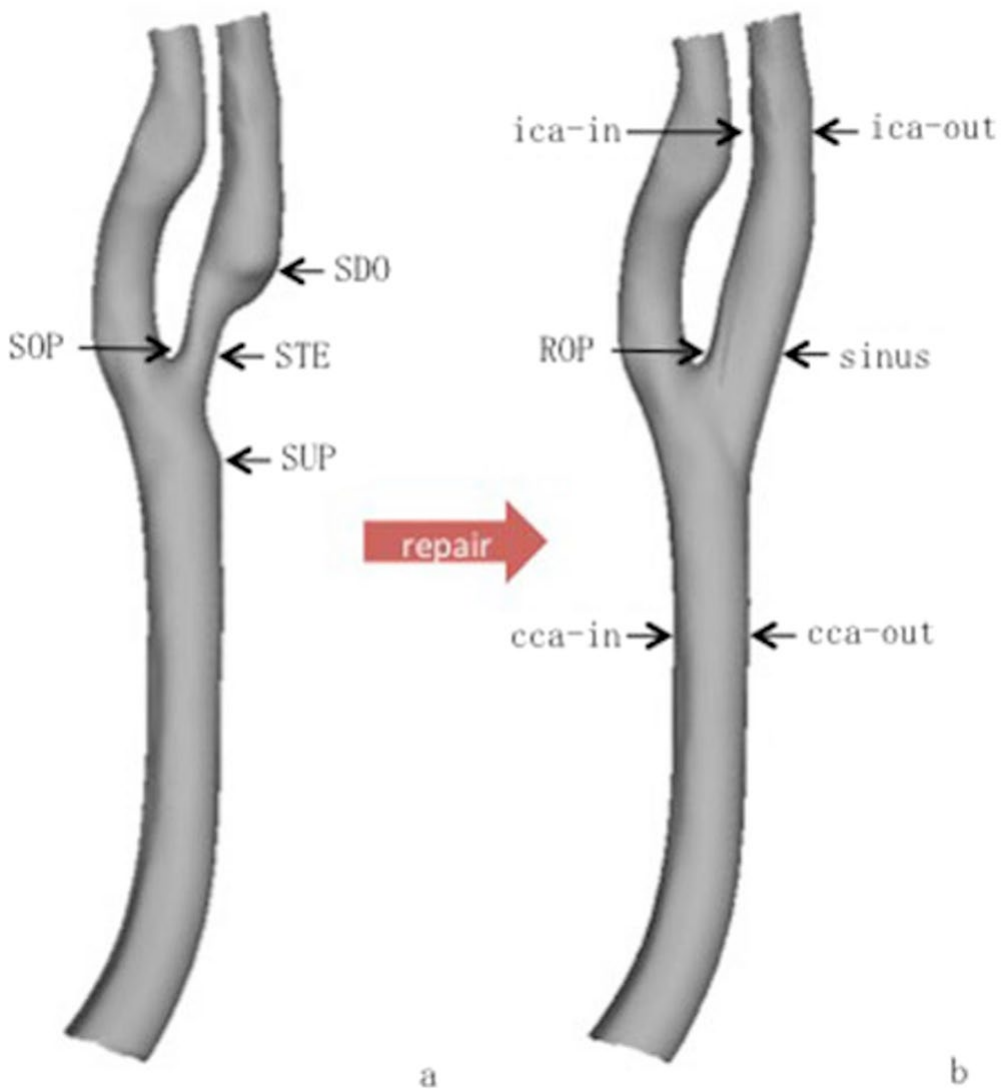
Regions of interest in a stenotic carotid artery (**a**) and stenosis-repaired carotid artery (**b**). SUP: upstream of stenosis, which is normal carotid area proximal close to stenosis; STE: stenotic segment; SOP: ICA opposite to STE; SDO: downstream of stenosis, which is normal carotid area distal close to stenosis. cca-out: outer lateral at CCA level; cca-in: inner lateral at CCA level; ROP: ICA opposite to sinus; ica-out: outer lateral at ICA level; ica-in: inner lateral at ICA level. CCA level and ICA level were located three vessel diameters proximal and distal to the bifurcation, respectively.

Six regions of interest (ROIs) in stenosis-repaired and normal carotid arteries were obtained for analysis, including five atherosclerosis-free areas [internal carotid artery (ICA) opposite to sinus (ROP) and outer and inner lateral at CCA and ICA level] and sinus. CCA level and ICA level were located at the three vessel diameters proximal and distal to the bifurcation, respectively^27^. Four ROIs in stenotic arteries were obtained for analysis, including the maximal stenotic segment (STE), the ICA opposite to STE (SOP), and upstream and downstream of stenosis that was located in the outer lateral of normal carotid areas proximal and distal to the plaque (SUP and SDO, respectively). The hemodynamic parameters of WSS and velocity were obtained. Continuous variables were reported as median (P25-P75). Wilcoxon rank sum test was used to analyze the WSS and velocity. P values <0.05 were considered significant. The streamlines and vectors of the flow illustrated the flow pattern (Fig. 4).

### Data Availability Statement

All data generated or analyzed during this study are included in this published article.
